# Formation of the hindgut cuticular lining during embryonic development of *Porcellio scaber* (Crustacea, Isopoda)

**DOI:** 10.3897/zookeys.515.9468

**Published:** 2015-07-30

**Authors:** Polona Mrak, Urban Bogataj, Jasna Štrus, Nada Žnidaršič

**Affiliations:** 1 Department of Biology, Biotechnical Faculty, University of Ljubljana, Večna pot 111, 1000 Ljubljana, Slovenia

**Keywords:** Development, digestive system, cuticle, extracellular matrix, embryo

## Abstract

The hindgut and foregut in terrestrial isopod crustaceans are ectodermal parts of the digestive system and are lined by cuticle, an apical extracellular matrix secreted by epithelial cells. Morphogenesis of the digestive system was reported in previous studies, but differentiation of the gut cuticle was not followed in detail. This study is focused on ultrastructural analyses of hindgut apical matrices and cuticle in selected intramarsupial developmental stages of the terrestrial isopod *Porcellio scaber* in comparison to adult animals to obtain data on the hindgut cuticular lining differentiation. Our results show that in late embryos of stages 16 and 18 the apical matrix in the hindgut consists of loose material overlaid by a thin intensely ruffled electron dense lamina facing the lumen. The ultrastructural resemblance to the embryonic epidermal matrices described in several arthropods suggests a common principle in chitinous matrix differentiation. The hindgut matrix in the prehatching embryo of stage 19 shows characteristics of the hindgut cuticle, specifically alignment to the apical epithelial surface and a prominent electron dense layer of epicuticle. In the preceding embryonic stage – stage 18 – an electron dense lamina, closely apposed to the apical cell membrane, is evident and is considered as the first epicuticle formation. In marsupial mancae the advanced features of the hindgut cuticle and epithelium are evident: a more prominent epicuticular layer, formation of cuticular spines and an extensive apical labyrinth. In comparison to the hindgut cuticle of adults, the hindgut cuticle of marsupial manca and in particular the electron dense epicuticular layer are much thinner and the difference between cuticle architecture in the anterior chamber and in the papillate region is not yet distinguishable. Differences from the hindgut cuticle in adults imply not fully developed structure and function of the hindgut cuticle in marsupial manca, possibly related also to different environments, as mancae develop in marsupial fluid. Bacteria, evenly distributed within the homogenous electron dense material in the hindgut lumen, were observed only in one specimen of early marsupial manca. The morphological features of gut cuticle renewal are evident in the late marsupial mancae, and are similar to those observed in the exoskeleton.

## Introduction

Epidermal body surfaces and ectodermal parts of the digestive system in crustaceans are covered by cuticle. The exoskeletal cuticle and the digestive tract cuticle are both chitin-based apical matrices, but differ in ultrastructural organization and functions. Exoskeletal cuticle in terrestrial isopod crustaceans is organized in three principal horizontal regions: epicuticle, exocuticle and endocuticle, that differ in the ultrastructural architecture and composition (Price and Holdich 1980, Compere 1990, Štrus and Compere 1996, Ziegler 1997, Štrus and Blejec 2001, Hild et al. 2008, 2009, Vittori and Štrus 2014). Epicuticle is the outermost thin layer, which is not mineralized and is composed predominantly of lipoproteins. The detailed ultrastructure reveals that epicuticle consists of an outer epicuticle, composed of several thin sublayers, and an inner epicuticle. Exo- and endocuticle are mineralized layers and display ''lamellae'', a pattern appearing due to helicoidal arrangement of chitin-protein fibers. The mineral component of tergite cuticle consists of calcite and magnesium-calcite, limited to the exocuticle, and amorphous calcium carbonate and amorphous calcium phosphate in the endocuticle (Hild et al. 2008, 2009, Neues et al. 2011, Seidl et al. 2011, Luquet 2012). Formation of the new exoskeletal cuticle has been extensively studied in molting adult crustaceans, including isopods (Price and Holdich 1980, Compere 1990, Štrus and Compere 1996, Ziegler 1997, Štrus and Blejec 2001, Vittori et al. 2012). Exoskeletal cuticle during early ontogenetic development of crustaceans was examined previously by *in vivo* or histological observations in decapods, branchiopods and amphipods (Freeman and Costlow 1980, Anger 1983, Snyder and Chang 1986, Belk 1987, Helluy and Beltz 1991). Several early ultrastructural studies, probing embryonic surface matrices, were made in branchiopods, isopods and decapods (Morris and Afzelius 1967, Goudeau 1976, Goudeau and Lachaise 1983, Glas et al. 1997). Recent studies, focusing primarily on the ultrastructural aspect of exoskeletal cuticle differentiation during embryonic development, refer mostly to the insect cuticle (Konopova and Zrzavy 2005, Moussian et al. 2006, Moussian 2010), and were performed also in the aquatic amphipod *Parhyale hawaiensis* (Havemann et al. 2008) and in *Porcellio scaber* (Mrak et al. 2014). As revealed in both these studies, the early extracellular matrix of epidermal cells appears as a thin delicate sheet of material. Later, the embryonic epidermis secretes a substantial matrix, termed embryonic cuticle or precuticular matrix, which is structurally different from the crustacean exoskeletal cuticle. A cuticular matrix with the general characteristics of crustacean exoskeletal cuticle is formed in the last stages of embryonic development. The new cuticle formation in the subsequent marsupial manca stages and the renewal of the exoskeleton in late-stage marsupial mancae have been described by Mrak et al. (2012, 2014). Larvae, termed postmarsupial mancae, are released from the marsupium and develop in the external environment until the juvenile stage (Tomescu and Craciun 1987, Brum and Araujo 2007, Montesanto et al. 2012).

The structure, composition and formation of the cuticle in the digestive system of crustaceans have not been precisely characterized. The digestive system of isopod crustaceans consists of the ectodermal foregut and hindgut and endodermal digestive glands, named also hepatopancreas or midgut glands (Hames and Hopkin 1989, Wägele 1992, Štrus et al. 1995). The two main regions of the foregut are the esophagus and stomach, the latter termed also the proventriculus. In the hindgut three morphologically and functionally distinct sections are distinguished: anterior chamber, papillate region and rectum. A short midgut situated between the foregut and hindgut, connected to the hepatopancreas, was described in amphibious species of the family Ligiidae (Štrus and Drašlar 1988, Štrus et al. 1995). The ectodermal digestive tract epithelium is apically lined by cuticle and performs specific functions in certain gut regions, including grinding, filtration, transport and absorption of food, and ion transport (Hryniewiecka-Szyfter and Storch 1986, Storch 1987, Storch and Štrus 1989). In the early studies of the digestive tract epithelium in isopods, two layers of the gut cuticle were distinguished, characterized as epicuticle and endocuticle (Vernon et al. 1974, Palackal et al. 1984) and cuticular spines were observed, covering the majority of the gut surface (Storch and Štrus 1989). In the stomach of terrestrial isopods complex cuticular structures were described, forming elaborate masticatory and filtering devices (Storch and Štrus 1989).

Differentiation of the gut cuticle during embryonic development is a poorly understood issue. Embryos of terrestrial isopods develop in the aqueous environment of the marsupium, a fluid-filled brood pouch on the ventral side of the female body. Intramarsupial development of *Porcellio scaber* lasts about 35 days under laboratory conditions and includes embryonic development, from fertilized egg to the early-stage embryo, the mid-stage embryo and the late-stage embryo, and development of the marsupial larva manca until release to the external environment (Milatovič et al. 2010). Wolff (2009) and Milatovič et al. (2010) have defined a staging system, describing twenty developmental stages, based on morphological characteristics of embryos and marsupial mancae in *Porcellio scaber*. Concerning digestive system development in embryos and marsupial mancae, the following morphological features of ectodermal digestive tract formation have been reported: (i) the invaginated stomodeum is discernible and hindgut invagination is evident between pleomere 6 and telson in mid-stage embryos (stage 6), (ii) the foregut and hindgut are fused in late embryos (stage 16), (iii) the cuticular masticatory apparatus, primary and secondary filters are present in the stomach of late embryos (stage 18), (iv) the hindgut is clearly partitioned into the anterior chamber and papillate region in late embryos (stage 18) and (v) the alimentary canal with a pronounced typhlosole is fully developed in marsupial mancae (Štrus et al. 2008, Milatovič et al. 2010). Cuticle formation in the digestive system was not followed in detail in these studies.

In our study, ultrastructure of the hindgut cuticular lining in *Porcellio scaber* embryos and marsupial mancae was characterized and compared to the hindgut cuticular lining of adults. We report on the hindgut cuticle differentiation from the structural viewpoint and discuss the results with respect to the differentiation of exoskeletal cuticle during intramarsupial development. Our aim was to describe the details of cuticle formation in different embryonic stages and to establish whether the hindgut cuticle of the emerging mancae is already fully developed.

## Methods

Specimens of *Porcellio scaber* Latreille, 1804 (Crustacea: Isopoda) were maintained and bred in a laboratory culture, in soil and leaf litter, at 25 °C, high relative humidity and at 12-h light/12-h dark cycle. Three adult animals without any external signs of molting were selected from the culture and anaesthetized by cooling. The guts were isolated, rinsed in physiological solution (0.9% NaCl), cleaned and divided transversely into three portions. Samples were fixed in 2.5% glutaraldehyde in 0.1 M Hepes buffer (pH 7.2). After washing with 0.1 M Hepes buffer, the samples were postfixed in 1% osmium tetroxide for 2 h and washed again in the buffer.

Embryos and mancae were isolated from the marsupia of gravid *Porcellio scaber* females and the stages of embryos were identified according to the existing staging system (Milatovič et al. 2010). The marsupial mancae were classified in accordance with the staging system reported by Mrak et al. (2012), distinguishing three sequential marsupial manca stages. In this study, three stages of late embryos (stage 16, stage 18 and stage 19) and two stages of marsupial mancae (early marsupial manca and late marsupial manca) were analysed. Specimens of embryos and mancae were fixed in 2.5% glutaraldehyde in 0.1 M cacodylate buffer (pH 7.2). Prior to fixation, the egg envelopes of embryos were either carefully perforated with a thin needle or removed. After fixation, specimens were washed with the buffer, postfixed in 1% osmium tetroxide for 2 h and washed again in the buffer.

All samples were then dehydrated using ethanol and/or absolute acetone and were embedded in Agar 100 or Spurr's resin. Polymerization of the resin was performed at 60 °C for 48 h in embedding molds. Semithin and ultrathin sections were made with a Reichert Ultracut S ultramicrotome (Leica), using a glass or a diamond knife, respectively. Semithin sections were stained with Azure II – Methylene Blue and imaged with a Zeiss AxioImager Z.1 light microscope, equipped with a HRC Axiocam camera using Axiovision software. Ultrathin sections were contrasted with 4% uranyl acetate and 10% lead citrate and examined with a Philips CM100 transmission electron microscope. Images were recorded with BioScan 792 and Orius 200 (Gatan) cameras using Digital Micrograph software.

## Results

### Cuticular lining in the hindgut of adults

**Figure 1.**
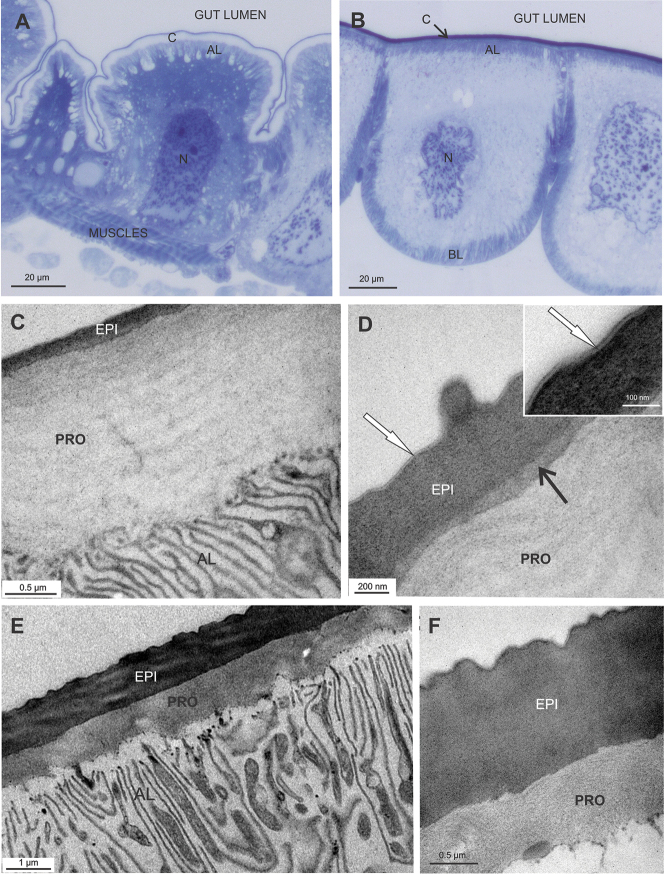
Hindgut epithelium and cuticle in *Porcellio scaber* adults. **A** Semithin section of the hindgut anterior chamber. Gut cells protrude apically into the gut lumen. The apical membrane forms an apical labyrinth (AL), that is covered with the cuticle (C). N – nucleus of gut cell **B** Semithin section of the hindgut papillate region. Gut cells bulge basally into the hemocoel. Apical and basal labyrinths (AL, BL) are evident. Cuticle covers apical cell surface (C). N – nucleus of gut cell **C, D** Ultrastructure of the cuticle in anterior chamber. The cuticle is composed of thin electron dense epicuticle (EPI) and much thicker ''lamellated'' electron lucent procuticle (PRO). Several thin sublayers are discernible in the outermost part of the epicuticle (**D** inset - white →). A layer of medium electron density is visible between the epi- and procuticle (**D** - black →). A cuticular spine is present on the cuticle surface **E, F** Ultrastructure of the gut cuticle in papillate region. Epicuticle (EPI) and procuticle (PRO) are about the same thickness. Both are composed of morphologically homogenous matrix. Abundant mitochondria are observed closely to the membranes of the apical labyrinth (AL) **F** Several thin sublayers in the outermost region of the epicuticle are visible.

The hindgut epithelium consists of large cells, apically covered with cuticular matrix and subtended by muscle cells (Fig. 1). In the anterior chamber the apical parts of epithelial cells bulge into the gut lumen, while in the papillate region the cells protrude basally into the hemocoel, which accounts for the 'papillate' morphology of this region (Fig. 1A, B). The hindgut cuticle of adults is approximately 2 µm thick and consists of an electron dense epicuticle and an electron lucent procuticle (Fig. 1C–F). The major part of the epicuticle consists of a homogenous electron dense matrix. As revealed by detailed ultrastructure, two to three thin sublayers are discernible in the outermost part of the epicuticle (Fig. 1D inset). In the anterior chamber a thin layer of medium density is apparent between the epicuticle and procuticle (Fig. 1C, D). The procuticle in the anterior chamber is much thicker than the epicuticle, with the ratio of the thicknesses around 5:1. The procuticle displays ''lamellae'', appearing due to helicoidal arrangement of chitin-protein fibers (Fig. 1C). Short cuticular spines are present on the cuticle surface (Fig. 1D). In the papillate region of the hindgut, the thickness of epicuticle is similar to that of the procuticle and no pattern of the chitin-protein fibers arrangement can be discerned in the procuticle (Fig. 1E, F). In both hindgut regions the apical plasma membrane of epithelial cells is differentiated in a distinctive apical labyrinth, with abundant mitochondria apposed to the membranes (Fig. 1C, E).

### Apical matrix of the hindgut cells in late embryos

**Figure 2.**
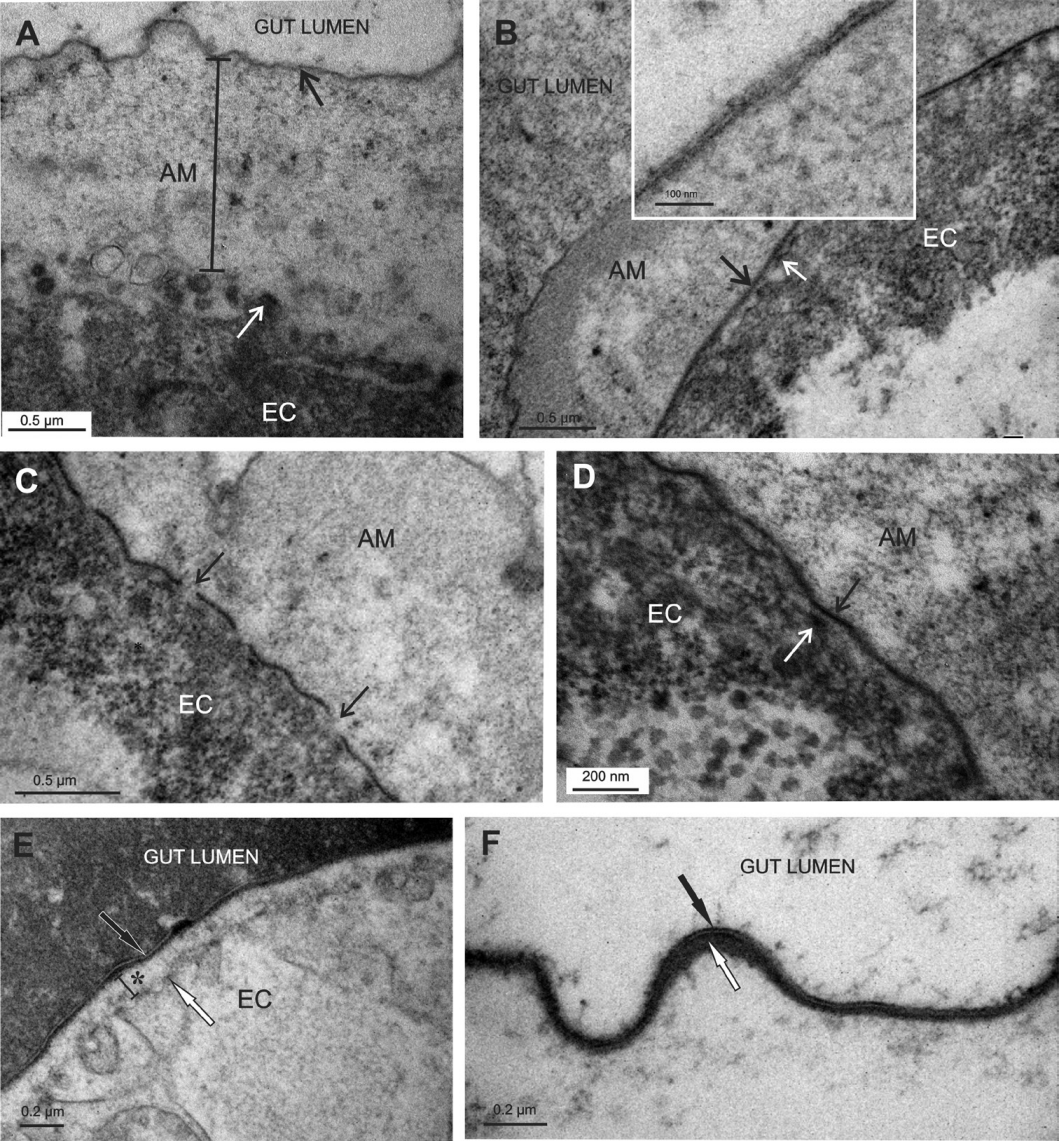
Apical matrices in the hindgut of *Porcellio scaber* late embryos. EC - epithelial cell **A** The hindgut cells (EC) in stage 16 embryos are covered by a substantial apical matrix with intensely ruffled surface (AM). The matrix consists of an electron dense lamina (black →) and underlying more electron lucent homogenous material. The apical membrane displays irregularly arranged protrusions (white →) **B, C, D** In the stage 18 embryos the apical matrix of the hindgut (AM) is extensive. The surface lamina covers the matrix, which displays a distal region of medium density and a proximal lucent region. The lamina of this matrix is trilayered (**B** inset). A new electron dense lamina (**B, D** - black →) is evident above the apical membrane protrusions (**B, D** - white →). The new lamina is mostly continuous, though in some regions it still appears in fragments (**C** - black →) **E, F** In the prehatching embryo of stage 19 the hindgut apical matrix consists of a distal trilayered lamina (black →), an electron dense material, accumulating underneath the lamina (**F** - white →) and underlying lucent material (**E** - *). Microvilli-like protrusions of the apical plasma membrane are evident (**E** - white →). The gut lumen is filled with homogenous material.

The apical matrix of the hindgut cells in stage 16 late embryos consists of intensely ruffled electron dense lamina and more lucent homogenous material underneath. The apical surface of the epithelial cells frequently forms irregularly arranged membrane protrusions with electron dense plaques (Fig. 2A).

In embryos of the stage 18 the hindgut apical matrix consists of an intensely ruffled thin lamina on the surface and matrix underneath, which is more dense in comparison to stage 16 embryos (Fig. 2B, C, D). The surface lamina is three-layered, consisting of two electron dense sheets with a lucent sheet between them (Fig. 2B inset). The matrix under the lamina displays two regions: a distal region of medium density, and a proximal lucent region. Apical plasma membrane forms regularly arranged shallow protrusions with electron dense apical plaques. Closely apposed to these protrusions is another electron dense lamina, evident all along the hindgut, reflecting secretion of the new cuticle. The new lamina does not show any distinct substructure (Fig. 2D). It is formed from fragments, which are observed in some regions (Fig. 2C), but it is mostly evident as a continuous layer along the hindgut in this stage (Fig. 2B, D). Spine-like surface structures were not apparent.

In prehatching late embryos of stage 19, the apical matrix does not display a ruffled outline, but in general follows the apical gut surface. In some regions detachment of the matrix from the epithelium was observed. The hindgut matrix in this stage is composed of an electron dense lamina facing the hindgut lumen and underlying lucent material. The lamina is thicker in this stage than in the previous embryonic stages and consists of three layers (Fig. 2E, F). In addition, electron dense material of about 20 nm in thickness is accumulated underneath the lamina. This accumulation, seen in the cuticular matrix of the hindgut in adults, is first observed in this stage. The apical plasma membrane of the hindgut cells forms microvilli-like protrusions (Fig. 2E).

### Cuticular matrix in the hindgut of marsupial mancae

**Figure 3.**
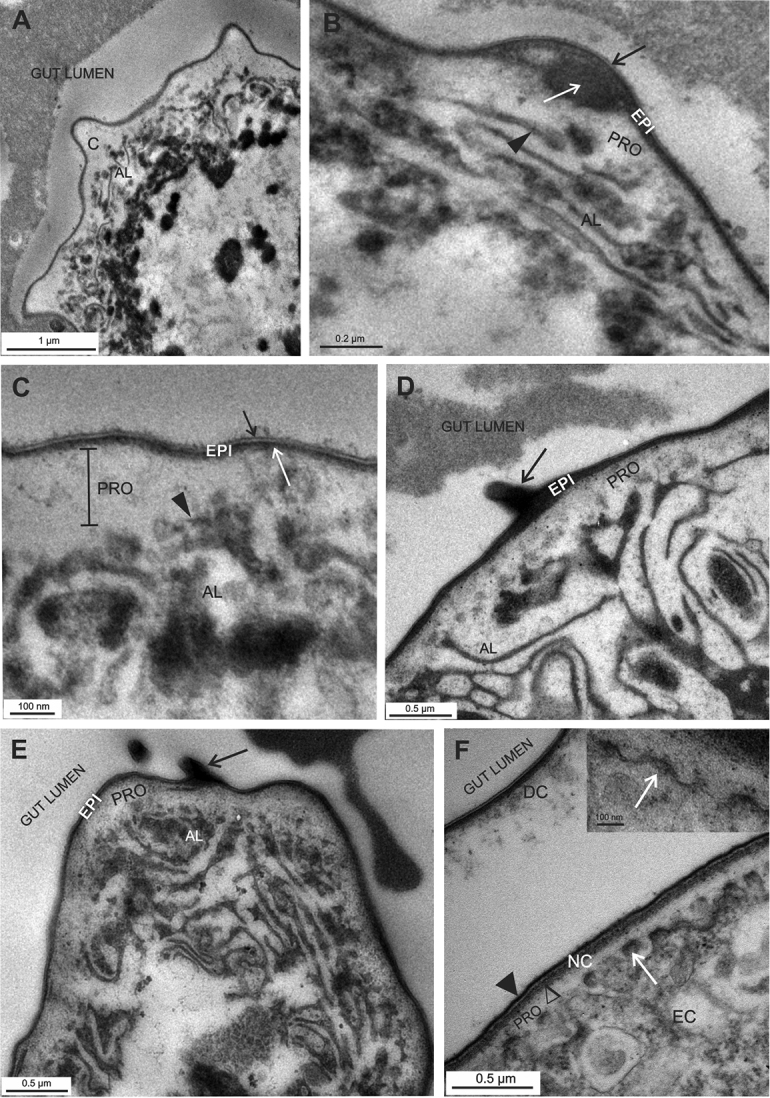
Cuticle in the hindgut of *Porcellio scaber* marsupial mancae. EC - epithelial cell, PRO – procuticle, EPI – epicuticle, AL – apical labyrinth. **A, B, C** The hindgut cuticle (**C**) in early marsupial manca with the outer epicuticle and the inner procuticle. The epicuticle (EPI) consists of the outermost trilayered lamina (**B, C** - black →) and electron dense material underneath (**C** - white →). The procuticle (PRO) contains homogenous electron lucent material. Bulges of the cuticle are observed, some include electron dense material (**B** white →). Apical plasma membrane is intensely invaginated (**B, C** – ►) and forms apical labyrinth (AL) **D, E** The hindgut cuticle in late marsupial manca in the anterior chamber (**D**) and in the papillate region (**E**). Electron dense material is prominent under the trilayered lamina of the epicuticle. Cuticular spines are evident (black →) **F** Hindgut cuticle renewal in late marsupial manca - degradation and detachment of the old cuticle (DC) and formation of the new cuticle (NC) on the plasma membrane protrusions (white →). The new cuticle consists of an electron dense lamina (►), an electron dense material accumulating underneath (∆) and an inner electron lucent homogenous procuticle (PRO) **F** inset: Protrusions of the apical plasma membrane (white →) display electron dense tips – plaques – and are covered with an electron dense material.

The luminal side of the hindgut epithelium of marsupial mancae is covered with cuticle, aligned to the surface of cells (Fig. 3) and consisting of epicuticle with a thin trilayered lamina and electron dense material underneath and procuticle, a thick homogenous lucent layer (Fig. 3B–E). No pattern of helicoidally arranged chitin-protein fibers is discernible in the procuticle. The apical plasma membrane is intensely invaginated, forming a labyrinth (Fig. 3A–E).

In early marsupial mancae the electron dense material under the trilayered lamina of the epicuticle is slightly more abundant than in stage 19 prehatching embryo (Fig. 3B, C). In some regions the cuticle protrudes into the gut lumen and electron dense material often accumulates in this bulge (Fig. 3A, B). In late marsupial mancae the electron dense material of the epicuticle is around 50 nm thick, which is slightly thicker than that in earlier larvae but still considerably thinner than in the cuticle of adults. Cuticular spines are evident on the cuticle surface in both the anterior chamber and the papillate region (Fig. 3D, E). The apical plasma membrane forms a prominent apical labyrinth (Fig. 3D, E). In addition, the morphological characteristics of cuticle renewal are observed in some specimens (Fig. 3F). Following apolysis, the old cuticle is detached from the epithelium and partly degraded, particularly in the basal part. In the formed ecdysial space the new cuticle formation takes place on the apical plasma membrane protrusions (Fig. 3F, 3F inset). The new cuticle is composed of two distinctive layers of similar thickness, the electron dense epicuticle and the inner homogenous procuticle (Fig. 3F).

**Figure 4.**
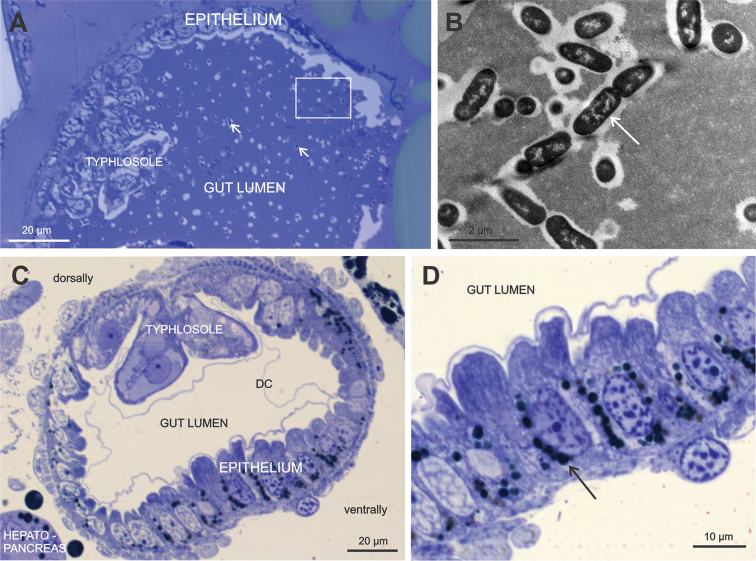
**A, B** The gut lumen contents in the early marsupial manca of *Porcellio scaber* includes homogenous material with evenly distributed bacteria (white →). A higher magnification of the squared area in the image **A** is shown in the image **B** Bacteria are rod-shaped, contain electron dense cytoplasm and are surrounded by lucent spaces. **C, D** Empty gut lumen, observed in the late marsupial manca. The cuticle is in most regions considerably detached from the epithelium (DC). The epithelial cells are ventrally more prismatic and dorsally more isodiametric. A higher magnification of the ventral gut cells in the image **D** reveals basally accumulated lipid droplets (black →).

In some examined specimens the gut lumen was empty, although in most marsupial mancae homogenous gut contents were observed (Fig. 4). In one specimen of the early marsupial manca, numerous bacteria were evident within the homogenous contents of the hindgut lumen (Fig. 4A, B). These bacteria have an electron dense cytoplasm, are rod-shaped, about 0.5 µm × 1.5–2 µm in size, and always surrounded by lucent spaces. The hindgut epithelial cells of the late marsupial manca contain lipid droplets, which are accumulated mostly in the basal part of the cytoplasm and are more abundant in the ventral gut epithelium (Fig. 4C, D). Differences in hindgut cell shapes are also observed between the dorsal and ventral epithelia, with the ventral cells being prismatic and apically bulging into the lumen and the dorsal cells by the typhlosole showing a more isodiametric shape (Fig. 4C).

## Discussion

Morphogenesis of the digestive system was previously studied in isopod crustacean *Porcellio scaber* (Štrus et al. 2008, Milatovič et al. 2010). Development of the cuticular lining in the ectodermal digestive tract is also essential for gut function, but gut cuticle formation was not studied in detail. In this study the hindgut cuticle differentiation in *Porcellio scaber* embryos and marsupial mancae is described and discussed with respect to the hindgut cuticular lining in adults and with respect to exoskeletal cuticle differentiation.

The hindgut cuticular lining in adults of *Porcellio scaber* is approximately ten times thinner than the exoskeletal cuticle. We show here that in the anterior chamber of the hindgut the procuticle is much thicker than the epicuticle, the ratio of the thicknesses being approximately 5:1. In the hindgut papillate region the epicuticle is about the same thickness as the procuticle and several times thicker than the epicuticle in the anterior chamber. The procuticle of the anterior chamber displays ''lamellae'' similar to those in the exoskeletal cuticle that appear due to the helicoidally arranged chitin-protein fibers. In contrast, the procuticle in the papillate region is morphologically homogenous. Two hindgut regions, anterior chamber and papillate region, are known to perform specific functions (Hames and Hopkin 1989) and are also characterized by specific ultrastructural features of the gut cuticle, as we show here.

The apical matrix secreted by hindgut cells in late embryos of stages 16 and 18 consists of loose material overlaid by an intensely ruffled electron dense lamina and thus resembles the epidermal precuticular matrix that is formed prior to exoskeletal cuticle during embryonic development (Mrak et al. 2014). Thickness of this hindgut matrix is in the same range as that of the epidermal precuticular matrix. Hindgut and epidermal matrices that are structurally similar in these embryonic stages, differentiate during further development into two cuticles of considerably different structure and thickness. The structural resemblance of embryonic gut and epidermal precuticular matrices in this species to the embryonic epidermal matrices preceding cuticle formation in insects and other crustaceans (Goudeau 1976, Goudeau and Lachaise 1983, Glas et al. 1997, Moussian et al. 2006, Konopova and Zrzavy 2005, Havemann et al. 2008) implies a more common principle in differentiation of chitinous matrices. The embryonic gut matrix is structurally different from the gut cuticle in adults, indicating that it does not yet perform all the specific functions of the fully differentiated gut cuticle in the adults. Still, this matrix may function as a protective barrier or may participate in a transport regulation. The intensely ruffled surface and loose structure suggest that the gut matrix in late embryos may serve to accommodate changes related to growth and bending of the embryo. In addition, the connection of the embryo with the osmoregulatory dorsal organ is lost at this period of embryogenesis, and this probably causes changes in the osmoregulatory capacity of the embryo (Milatovič et al. 2010).

The hindgut matrix in prehatching stage 19 embryos consists of a trilayered electron dense lamina, subjacent electron dense material and the innermost lucent layer. We consider this matrix a hindgut cuticle as it strictly follows the apical epithelial surface and includes a prominent layer of electron dense material below the lamina, characterizing the gut epicuticle. The apical membrane protrusions in some regions suggest the secretion of the procuticle components. In this stage the procuticle in the hindgut is not sublayered as it is in fully formed cuticle, while the exoskeletal cuticle already displays ultrastructure similar to that in adults (Mrak et al. 2014). As an electron dense lamina apposed to the apical membrane of the hindgut cells was observed in the preceding developmental stage – stage 18 embryo – we consider this the first epicuticle formation. The results thus indicate that the cuticle is secreted prior to the shedding of the early apical matrix. The lamina is still discontinuous in some regions and does not form any surface structures. In this developmental stage (stage 18) the first exoskeletal cuticle formation has been described and observed in all regions as a continuous cuticular matrix, also forming epicuticular scales (Mrak et al. 2014). The early phase of exoskeletal cuticle formation during embryogenesis of *Drosophila melanogaster* has been characterized by the presence of fragments of the outer epicuticle, which is described in insects as an ''envelope''. In later development the gaps between the fragments are closed, forming a continuous envelope (Moussian et al. 2006). Our results indicate that the initiation of hindgut cuticle formation similarly appears in fragments, which is consistent with exoskeletal cuticle formation described in other arthropods, for example in *Drosophila melanogaster* (Moussian et al. 2006).

The hindgut cuticular lining in marsupial manca, with the electron lucent procuticle and overlying electron dense epicuticle, shows more similarities to the hindgut cuticle in adults. Advanced differentiation is evidenced by more prominent electron dense layer of the epicuticle and formation of cuticular spines. Extensive invaginations of the apical plasma membrane, forming a prominent apical labyrinth, suggest that hindgut epithelium is involved in transportive processes in mancae. The morphology of hindgut epithelium and cuticle in marsupial mancae implies that the specific functions of the hindgut in feeding are more developed. We have observed that in most examined mancae the gut lumen is filled with homogenous contents. Warburg (1994) also reports on intramarsupial cannibalism of marsupial mancae. Compared to the hindgut cuticle of adults, the cuticle in marsupial manca is five to twenty times thinner, the procuticle in the anterior chamber is not sublayered and the ratio of epicuticle to procuticle thicknesses is similar in different hindgut regions. These differences indicate that the hindgut cuticle in marsupial mancae does still not perform all functions of the gut cuticle in adults, which could be related to the aqueous environment of the marsupium, among other not exposing the animal to dissication. As described for the exoskeleton (Mrak et al. 2012, 2014), morphological characteristics of cuticle renewal in late marsupial mancae are observed also in the hindgut, indicating the renewal of the gut cuticle soon after release of mancae from the marsupium and subsequent molting of the mancae. Bacteria recorded in one specimen of an early marsupial manca were distributed evenly within the contents of the hindgut lumen and no specific attachments to the cuticular lining were observed. Kostanjšek et al. (2003) reported on autochthonous bacteria associated with the wall of the hindgut papillate region in adult *Porcellio scaber*. They described rod-like bacteria of 2.5 µm ± 1 µm in length and 200 nm ± 50 nm in width and filamentous microbes with lengths up to 6 µm, both specifically attached to the cuticular spines. Bacteria observed in our study are not attached to the hindgut cuticle and resemble neither the rod-like bacteria by size nor the filamentous microbes described by Kostanjšek et al. (2003).

## Conclusions

**Figure 5.**
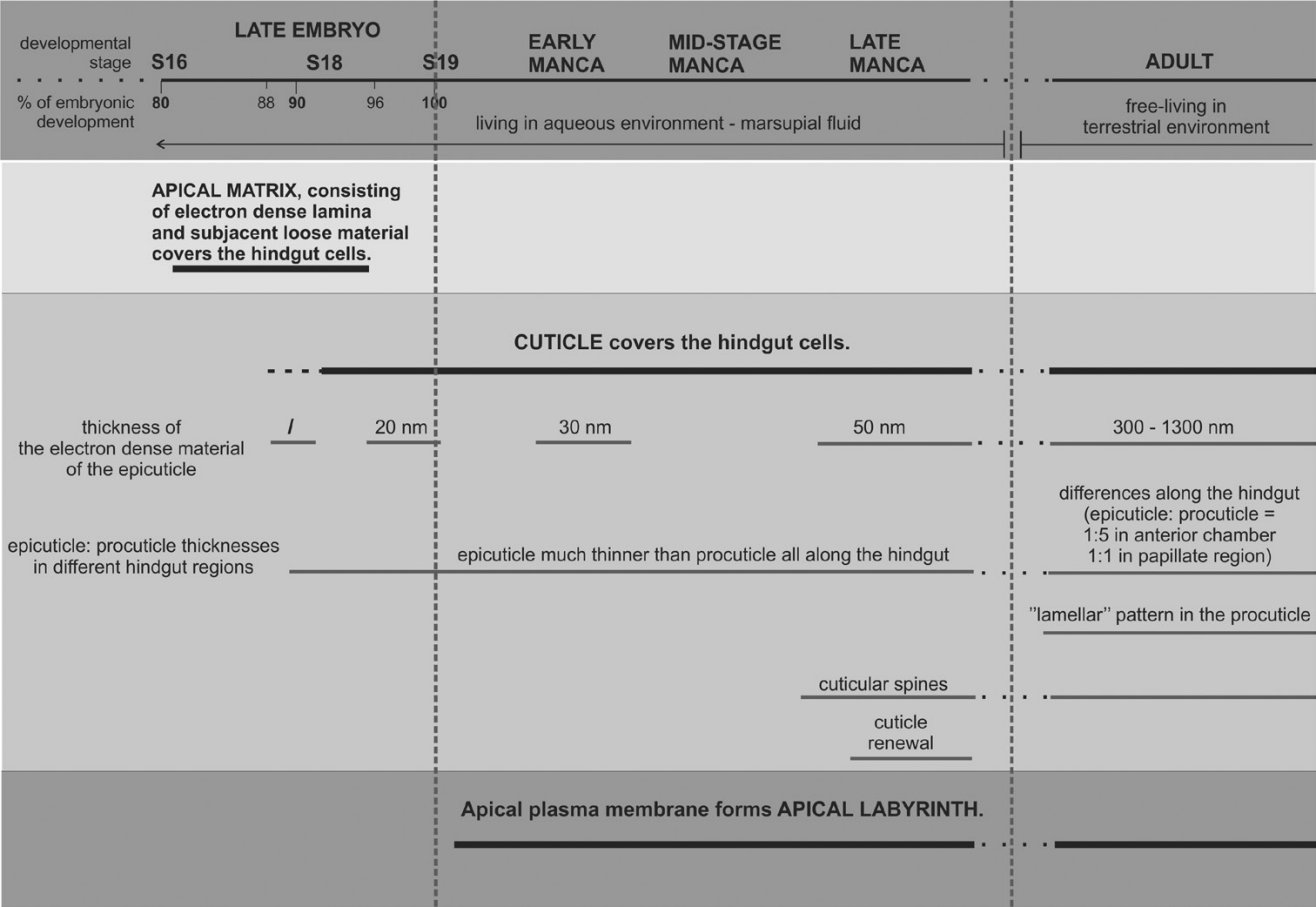
A schematic representation showing the ultrastructural characteristics of the hindgut apical matrices and epithelium during late intramarsupial development and in comparison to the hindgut cuticular lining of adult animals in *Porcellio scaber*. The axis represents the successive developmental stages and the percentage of embryonic development. The vertical dashed lines indicate the transition from embryonic to larval development and from larval development to adult stage. The thick horizontal lines represent presence of the individual feature in the certain stages. The specific features of the cuticle are indicated by the thin lines.

Ultrastructural characteristics of the hindgut cuticular lining and epithelium, according to the examined developmental stages of *Porcellio scaber* and in comparison to the hindgut of adults, are shown in a schematic representation in Figure 5. Identified by ultrastructural analysis, the early stages of hindgut apical matrix formation during embryonic development are similar to the early differentiation of epidermal matrices, reported in arthropods. This structural resemblance suggest similar underlying processes of chitinous cuticle differentiation. Structural difference from the hindgut cuticle in adults suggests that early hindgut apical matrices are not fully involved in the specialized functions of the cuticle.

In the advanced intramarsupial stages the hindgut cuticle is structurally more similar to the hindgut cuticle in adults, suggesting it progressively assumes its specific functions in protection and food processing. In the stage 19 prehatching embryo a hindgut cuticle is evident, characterized by alignment to the epithelial surface and a prominent electron dense layer of the epicuticle. An electron dense lamina in the preceding embryonic stage - stage 18 - is evident, considered as the first epicuticle formation. This is the same stage, in which the first exoskeletal cuticle formation has been observed.

In marsupial mancae further gut cuticle differentiation is evident, as formation of the cuticular spines and a conspicuous electron dense epicuticular layer. Compared to the gut cuticular lining of adults, the cuticle of marsupial mancae is thinner, the ultrastructure is not different along the length of the hindgut, the procuticle in the anterior chamber does not yet show any lamellae associated with chitin helicoids, and the electron dense layer of the epicuticle is thinner. These differences imply that the function of the hindgut cuticle in marsupial manca is not fully developed, possibly related also to different environments, as mancae develop in marsupial fluid. Synthesis of new cuticle prior to ecdysis is evident in both the exoskeleton and hindgut cuticle of late marsupial mancae.
